# Kenya’s “Fake Essay” Writers and the Light they Shine on Assumptions of Shadows in Knowledge Production

**DOI:** 10.1080/13696815.2021.1952405

**Published:** 2021-09-20

**Authors:** Patricia Kingori

**Affiliations:** The Wellcome Centre for Ethics and Humanities and the Ethox Centre, University of Oxford, UK

**Keywords:** Shadow Scholars, fake, Kenya

## Abstract

In this contribution to the special issue on Fakery in Africa, I examine the booming “fake essay” industry and draw on the role and perspectives increasingly occupied by of tens of thousands of young and highly-educated Kenyans. These so-called “Shadow Scholars” are part of a vast global online marketplace, an invisible knowledge production economy, where students and academics in the global North solicit and pay for their services in exchange for confidential and plagiarism-free essays, theses, dissertations, qualifications and publications. This article centres on descriptions of these writers as “shadows” as a means of complicating not only the most popular description of Africa in the global imagination – as existing in the shadow of an infinite number of different entities – but to challenge the notion of the shadow in relation to African knowledge production as being fake. It pays attention to the Kenyan writers’ protestations that their knowledge, experiences and labour are all real and that analogies with shadows reduce them and the impact of their work to something that is non-existent and not alive. From their perspective the term shadow is pejorative because it further reduces the intellectual contribution of Africans, presenting them as derivative.

## ABSTRACT IN SWAHILI

Katika mchango huu kwa suala maalum kuhusu Udanganyifu barani Afrika, mimi huchunguza tasnia ya “insha bandia” na haswa jukumu na msimamo wa makumi ya maelfu ya vijana wakenya na wenye elimu ya kiwango cha juu. Hawa wanaoitwa “Vivuli vya Wasomi” ni sehemu ya soko kubwa la mkondoni ulimwenguni, uchumi wa maarifa usiojulikana, ambapo wanafunzi na wasomi katika kaskazini mwa Ulimwengu wanaomba na kulipia huduma zao kwa malipo ya insha za wizi na usiri, tasnifu, ujuzi wa kitaaluma na machapisho. Utafiti huu unaangazia ufafanuzi wa waandishi hawa kama “Vivuli” kama njia ya kutatanisha sio tu maelezo maarufu zaidi ya Afrika pia katika fikra za ulimwengu, kama ilivyo katika kivuli cha idadi isiyo na mwisho ya vyombo tofauti, lakini kupinga wazo la kivuli kuhusiana na uzao wa maarifa ya Kiafrika kama bandia. Inatilia maanani maandamano ya waandishi wa Kenya kwamba maarifa, uzoefu na kazi zao zote ni za kweli na kwamba mlinganisho na vivuli hupunguza na athari ya kazi yao kwa kitu ambacho hakipo na sio hai. Kulingana na maoni yao, neno kivuli ni la kudharaulisha kwa sababu linapunguza zaidi mchango wa kiakili wa Waafrika, kuwaonyesha kama waigaji.

## Introduction

I do a lot on nursing … that means I have to do all the research, writing … I will have to understand … the concepts … 

I feel it’s wrong sometimes, though it pays the bills. Cause clearly as a nurse, you are dealing with patients, real and live patients.

So when you give me a paper to do on your behalf. This is my brain, you know, clearly you’ve not made the effort to just do your own research on whatever is needed. So you’re basically using my knowledge, my brain and you’re dealing with a patient who is … hmm I don’t know, it’s just wrong but we need the job. (Philippa, face-to-face interview, Nairobi, 2019)

This introductory quote was gained from an interview with Philippa^1^ in Nairobi, where we discussed her work. Philippa, a graduate of a prominent Kenyan university with a Master's in Public Health from a UK university, is, like tens of thousands of graduates in Kenya, paid to write secretly for someone else. Philippa’s role in the final product is made invisible and the person who purchases her work then becomes the named author of what she has produced. From nursing, medical and scientific assignments, to journal publications, theses and dissertations for students and academics mainly in the UK and US, writing for others is part of what has become a million-dollar “fake essay” industry (Nakweya 2020; Walker 2017). Nairobi is widely reported to be the epicentre of this global industry, with conservative estimates of over 40,000 known writers actively engaged in this practice, mostly undertaking work for international students in the global North (Mutwiri 2019; White 2016; Melia et al. 2019).

This phenomenon has multiple facets: the history of Kenya and how these practices have developed over time; the role of technology in promoting and supporting this industry; socio-economic and structural forces which have produced a surplus of highly educated but unemployed young people; geopolitics which render Kenyans unable to take up positions elsewhere because of visa restrictions; and the demand, coming from mainly the global North, for such services provide important contextual insights and are worthy of detailed discussions in their own right. At the same time, as Philippa’s quote suggests, there are also numerous ethical considerations implicated in this phenomenon. These vary by the type of work performed, and for whom, but also include how those who write for others may best be named such that their contribution to global knowledge production can be made visible and taken seriously. As an entry point into some of the complex and interwoven features of this phenomenon I would like to focus on the labels given to the young Kenyans like Philippa involved in the “fake essay” industry, and the ways in which these labels reflect how they are seen and want to be seen by the world.

## Shadow Scholars

One of the few first-hand accounts of what it feels like to write for others was produced by American college student Dave Tomar. In his 2012 book, *The Shadow Scholar: How I Made a Living Helping College Kids Cheat,* Tomar – a graduate of Rutgers University and an aspiring writer – details his decade-long experience as an American who was paid to write assignments for other American students. In one year alone, Tomar claimed to have written almost 5000 pages of scholarly literature including a PhD in Sociology, a Master’s degree in cognitive psychology and essays on history, cinema, labour relations, marketing, philosophy and, ironically, ethics. He describes a typical day in which he would:

Write a ten-page paper on AIDS in Africa; a six-pager on the Manson Family murders; a dozen pages on the connection between violent video games and chronic masturbation. Explain the wanton criminal behavior of the Bush administration; the brazen corruption of the Bulgarian government; the gross hysteria of the Salem witch trials. Whip up a quick reflection on the massive human suffering created in the impoverished former states of the collapsed Soviet Union; the psychological complexity of the Nuremberg Trials; the disturbing failure of Euro Disney to excite discerning Frenchmen. (Tomar 2012, 286)

Tomar is self-described as a Shadow Scholar. The term is derived from his experience of being asked to write a paper on Plato’s “Allegory of the Cave”. Plato’s allegory was considered by Tomar to be significant because it describes a group of people who are born chained to the ground in the back of a cave, with shackles binding them at the arms and neck. The only thing they can see is a wall just beyond the cave’s mouth. The fire behind them creates shadows on the wall and these shadows are the sole access they have to life beyond the cave and an indefinable knowledge of the shadowy individuals that walk by freely outside the cave. As Plato argues, “To them … the truth would be literally nothing but the shadows of the images” (Shorey 1963, 747). In Plato’s allegory, one man is freed from bondage and struck by a revelation that he at once fears and knows he must share with the others in the cave. At first, when any of them is liberated and compelled suddenly to stand up and turn his neck round and walk and look towards the light, he will suffer sharp pains; the glare will distress him, and he will be unable to see the realities of which in his former state he had seen the shadows; and then conceive someone saying to him, that what he saw before was an illusion, but that now, when he is approaching nearer to being and his eye is turned towards more real existence, he has a clearer vision.

Tomar claims that he was particularly moved by the idea of giving insights into the real existence of the education system and it was this which prompted him to discuss his experience of the “fake essay industry” as a so-called Shadow Scholar. From Tomar’s position, there are positive connotations associated with the idea of being a Shadow Scholar. They are free from the misconceptions of how the education system truly works and exists for those with money and power. The chains that prohibit the captives from leaving the cave in Plato’s allegory reflect their imprisonment in ignorance, as the chains hinder them from discovering the truth. The shadows thrown on the cave walls represent the superficial truth, which is the illusion that the captives observe in the cave. By extension, Shadow Scholars in the sense that it is used by Tomar cannot be seen by those who are ignorant of their existence. There is also the sense in which Tomar is keen to align his experience with that of one of the most well-known Greek philosophers to impress on the reader that he is erudite and knowledgeable in a range of academic disciplines.

To date, Tomar’s work is one of the most detailed and influential autobiographies in the field. Almost ten years after *The Shadow Scholar* was published it remains important for shedding light on the perspectives of those involved in writing assignments and publications for others as part of the “fake essay” industry. However, increasingly, its reach has extended beyond American contexts to speak to African, and in particular Kenyan, writers as the term “Shadow Scholar” is now used to describe writers such as Philippa and a phenomena which is now an industrial global enterprise involving the labour of Africans on an unprecedented scale.

## Africa in the Shadow

A search of the keywords “Shadow” AND “Africa” yields thousands of academic publications and books from disciplines as diverse as agriculture to zoology. In fact, it is the most common descriptor of Africa and its economy, politics and daily life, its relationship to diseases such as malaria, HIV and AIDS and tuberculosis as well as its position in relation to development and the world order (e.g. Bajaj 2008). While hugely different, what all of these publications have in common is a consensus that the direction of the light on any given object places Africa in the shadow and that their tone is rarely positive in their descriptions of Africa. For the most part, Africa and the Africans within these texts are depicted as being belated, almost and not quite; always relational and subservient. Not only is Africa often described as being *in* the shadow but as authors such as James Ferguson’s (2006) work makes clear, the continent of Africa is often described as *the* *shadow* of global processes and practices. Writing in *Global Shadows* Ferguson seeks to problematise the ubiquitous use of analogies of Africa and shadows to depict the continent in terms of crisis, failure and intractable problems. By questioning this analogy, his work is important in allowing room for broader discussions of the analogy’s value in relation to the fake essay industry and the Kenyan writers involved in it.

## Are African Shadows the Same as Other Shadows?

In “Without a Shadow of Doubt” (2016), Kenyan writer and postcolonial theorist Ngũgĩ wa Thiong’o tells the story of two young Kenyan boys who set off to discover, through a series of endearing quasi-scientific studies, whether everybody’s shadow is black like theirs, or whether, as they suspect, white people have white shadows. The young boys in the story are in fact based on Ngũgĩ and his younger brother. In finding out that the shadows of all people, regardless of their race, are the same colour they also discover an important life lesson about the role of the observer’s perspective in the creation and appearance of shadows – depending on the vantage point and position of light the observer’s role is crucial in the formation of shadows. Years later, while attending an arts and film course in Europe, Ngũgĩ reflected on this experience and surmised that the “Light source, whether sun in the day or moon at night, or fire inside or outside a house, and the time, yes, the time or even the passage, through the window, or a crack in the wall, determined how shadows fell on the subject”. The light source and how the shadow falls on the subject are key to understanding how individual and collective perspectives produce knowledge and, to extend the argument made by Ngũgĩ further, to exploring what is real and what is fake.

Ngũgĩ’s reflections on the significance of shadows and their value as analogies to other areas of social life are important in this discussion of fakes and fakery in African spaces. In this article, Ngũgĩ’s discussion of shadows is useful in introducing ideas of positionality and the importance of the observer’s vantage point. When shadows are used as an analogy for racial equality they provide valuable ways to discredit ideas about racial superiority – all shadows are the same and no shadow is more real or fake than another. However, from the position of Kenyan participants in the so-called fake essay industry, the concept of the shadow and in particular that of the Shadow Scholar is considered by the Kenyans in question to be more problematic. In conversation, Sylvia made the point that the analogy with shadows has different connotations. She explained that:

The shadow is something that is not living, it has no impact. Being a shadow means that you are ineffective. If the shadow of a speeding truck passes across you it will have no impact on you but the truck will … From here in Kenya, we are having a big impact, we are helping thousands of people every year to get degrees, get tenure, get their research funding because we will write their grants, to keep their jobs, get promoted … many people sitting there in your offices are there because of our efforts. That’s a fact. They are there because of our contribution. Hey! In fact they are the shadows of us! [laughter]. (Sylvia, face-to-face interview, Nairobi, 2019)

In Kiswahili, to refer to someone as a “*kuvuli*” or a shadow, is to describe them as having not existed. “*Vivuli vya Wasomi*” (shadow of scholars) are scholars who do not or have not existed, who have no impact and are considered ineffective. The value of this quote is that it encourages us to pay greater attention to not only the genealogy of concepts used in African spaces but also the meaning and local interpretations of terms such as shadow and fake as descriptors, as they travel from the global North to the South. From Sylvia’s quote it is clear that this concept is the opposite of how these writers would like to be seen and moreover how they see themselves. In this way, paying attention to Ngũgĩ’s writing in *Decolonising the Mind* reminds us that language communicates “how people perceive themselves” and their “specific relationship to the world” (1986, 6).

For the Kenyan writers the name that they are given is more than an abstract exercise. What they are called and how they are seen have practical and real-world consequences. Whereas in Tomar’s use of the term “Shadow Scholar” he voluntarily places himself in the shadows, what the writers in Kenya are arguing against is a position where they have been forcibly placed in the shadows. Their contention with the term is that if they were to accept it, they would become complicit in the geopolitical forces and racial and national stereotypes about Africa and Africans which they believe conspire to make them invisible and considered as not real.

Sylvia’s comments also return us to the point made by Ngũgĩ (2016) in his discussion of shadows in asking from whose perspective does the shadow exist. Where is the observer when this phenomena is being viewed? These questions remind us of the importance of vantage point and position of light in relation to the creation and appearance of shadows. In exclaiming, “In fact they are the shadows of us!”, Sylvia draws attention to the perspectives of writers in Kenya who see themselves and their role in the so-called fake essay industry from the position of having knowledge and skills not possessed by the people who use their services.

## What’s in a Name?

This discussion of the shadow is pertinent to the discussion of fakes and fakery in African spaces more broadly. The concept of fakes and shadows when applied to this phenomena can be considered as a form of misrepresentation but also an example of epistemic violence (Castro-Gómez 2019). When Gayatri Spivak uses the term “epistemic violence” in her seminal text, “Can the Subaltern Speak?”, she was primarily referring to the silencing of marginalised groups such as “the illiterate peasantry”, “the tribals”, and the “lowest strata of the urban sub-proletariat” (Spivak 1998, 282–283). For Spivak the concept of “epistemic violence” relates to the ways that the hegemonic ideology acts to block the development of authentic experiential knowledge generated by subaltern classes’ everyday lives. While the Kenyan writers under examination in this article do not neatly fit with the economic or social populations described by Spivak, they are marginalised in more than one way, not least because in the global imagination they are seen, spoken of and assumed to be in need of education by those in the global North, rather than understood to be a population assisting students and academics all over the world in their education. Moreover, as Philippa outlined in the opening quote to this paper, Kenyan writers are increasingly employed to write on subjects which impact matters of health, science and medicine with implications for health systems in numerous countries. In this way, the significance of their role is far greater than described by Tomar in his account of the types of work, and the ethical implications involved, that he was employed to undertake.

While highly-educated, fluent in multiple languages and confident with technology, these Kenyan writers are not employees of any academic institutions (Kässi and Lehdonvirta 2018). Their position outside of academic institutions means that they are omitted from formal discussions of Kenya’s contribution to knowledge production and marginalised from gaining credit for their intellectual endeavours in such forums (Lancaster 2019). However, their views and positions are important to our understanding of the ways in which the young and ambitious make a life in contemporary African spaces, the role and value of education, and capturing their views can contribute to insights from a standpoint theory perspective (Pohlhaus 2002). In general, standpoint theories claim that the perspectives of subordinated social groups should be prioritised over all other accounts because their subordination, relative to the perspectives of the groups that dominate, gives them an epistemic advantage and unique insights into contested topics (Rolin 2009).

Philippa and Sylvia tell us that words such as shadow and fake misrepresent who they are in the world. George adds further insights from the standpoint of the Kenyan writers in stating:

I often wonder why given all the resources they have why they need us to write for them. We should be learning from them but they are learning from us … Us with our scarce resources are writing for some of the big universities. We use our brains. I think our brains are really superior. That’s the only explanation I can come up with … They have money and we have brains … That’s why for me I prefer to be called an academic or intellectual surrogate … yes we’re intellectual surrogates. (George, face-to-face interview, Nairobi, 2019)

From this standpoint, describing the Kenyan writers as in the shadow reveals a fundamental inversion of what they consider to be the case. Furthermore, describing these writers in such terms further distances them from the positions in the world they think that they should be occupying. Writing essays, dissertations and books for other people might bring these writers economic gains but they cannot disclose this form of employment when applying for formal, or what is considered legitimate, employment. In this way, writing for others lacks the opportunity for advancement and the chance to achieve the types of social status they believe their intellectual capacity should allow them to have. In contrast, Dave Tomar, who coined the term Shadow Scholar, has retired from the “fake essay” business. He is now a freelance journalist and has published for various news outlets including the *Huffington Post* and *New York Times*. Tomar has managed to successfully move out from the shadows and gain recognition for his intellectual capacity; this is the dream and aspiration for many of these writers in Kenya.

In the final section of this article I focus on how the writers describe themselves and how they would like to be described. In referring to themselves as “academic or intellectual surrogates” we are offered the opportunity to see these writers as they want to be seen. The term surrogate allows us to connect their position in the world with that of biological surrogates and from there to reconceptualise their contribution and their sacrifice in the knowledge production industry. As Philippa stressed in the opening quote: “This is my brain … you’re basically using my knowledge, my brain.” Such emphasis on their original intellectual contribution, and the intellectual property invested by intellectual surrogates, seeks to move them out of the realm of the fake and into a space where they can be recognised as highly skilled, resourceful and making a valued contribution in the world. Thus paying attention to the fake and descriptions of fakery in African spaces is insightful in numerous ways. In moving past superficial labels and in seeking to capture the standpoint perspective of those assigned to the category of the fake and described as shadows, we learn of the epistemic violence that occurs when such labels are imposed on those who have been marginalised. The Kenyan writers discussed here have drawn our attention to their role as intellectual surrogates, a position in the world that raises more questions of institutions in the global North than of those in African spaces.

## Conclusion

To fail to consider the labels we use in our descriptions of Africa and African spaces can cause epistemic violence. This claim is not new but the contribution here is in showing that even when we use seemingly innocuous and morally neutral terms such as “the shadow”, we can be complicit in reproducing a set of assumptions of where the real is situated. While less pejorative than terms such as “the fake”, descriptions of highly educated Africans as being partial misrepresents the size and scale of their contribution.

## Note

1 All names in the article are pseudonyms.
